# A roadmap for personalized medicine: the findings of the IC2PerMed project

**DOI:** 10.3389/fmed.2025.1572921

**Published:** 2025-07-30

**Authors:** Flavia Beccia, Francesco Andrea Causio, Marzia Di Marcantonio, Ilda Hoxhaj, Chiara Cadeddu, Melissa Campagno, Lena Schleicher, Carmen Fotino, Maike Tauchert, Marta Lomazzi, Lili Wang, Wenya Wang, Huiyao Huang, Walter Ricciardi, Stefania Boccia

**Affiliations:** ^1^Section of Hygiene, Department of Life Sciences and Public Health, Università Cattolica del Sacro Cuore, Rome, Italy; ^2^Faculty of Economics, Università Cattolica del Sacro Cuore, Rome, Italy; ^3^Department of Medical and Surgical Sciences, Fondazione Policlinico Universitario Agostino Gemelli IRCCS, Rome, Italy; ^4^Erasmus School of Health Policy and Management, Erasmus University Rotterdam, Rotterdam, Netherlands; ^5^G.A.C. Group - Innovation and Performance for Impact, Valbonne, France; ^6^Steinbeis Europa Zentrum (SEZ), Stuttgart, Germany; ^7^Fondazione Telethon, Milan, Italy; ^8^BBMRI-ERIC, Graz, Austria; ^9^World Federation of Public Health Associations (WFPHA), Geneva, Switzerland; ^10^Jingyi Alliance Clinical Application; BGI, Beijing, China; ^11^Center of Biotherapy, Beijing Tsinghua Changgang Hospital, Tsinghua University, Beijing, China; ^12^Academic Director, Clinical Trials Center of National Cancer Center, Beijing, China; ^13^Department of Woman and Child Health and Public Health, Fondazione Policlinico Universitario A. Gemelli IRCCS, Rome, Italy

**Keywords:** personalized medicine, roadmap, China, European Union, priorities

## Abstract

**Introduction:**

Personalized Medicine (PM) tailors prevention and treatment to individuals based on their unique characteristics. It has the potential to improve health outcomes and healthcare sustainability by optimizing resource allocation. Both the European Union (EU) and China have prioritized PM in their health strategies. The IC2PerMed project was established to foster collaboration between the EU and China by developing a joint PM roadmap.

**Methods:**

To assess the state of PM in the EU and China, the project conducted a comprehensive mapping of relevant policies, programs, funding mechanisms, and health ecosystems. Additionally, three Delphi surveys were carried out, identifying 65 priorities, which were synthesized into a set of strategic actions.

**Results:**

The main output of the project is a joint roadmap for implementing PM in the EU and China. The roadmap promotes best practice exchange and addresses potential barriers to PM adoption. It outlines structural actions including enhancing health literacy, fostering intersectoral and international collaboration, ongoing review of emerging technologies, and facilitating innovation market entry through needs assessment and Health Technology Assessment. Key enablers such as data interoperability and shared standards are highlighted, along with ethical, social, and regulatory considerations that are universally relevant to PM implementation.

**Discussion:**

A shared action plan can guide health policy and help policymakers understand the interconnection between healthcare, the economy, and society. By supporting international projects and investing in research and innovation, stakeholders can advance global healthcare.

## Introduction

Since the completion of the Human Genome Project two decades ago, significant advances in genomic medicine and the incorporation of genomic information in diagnostic, treatment, and reimbursement practices contributed to reshaping medical practice (1, 2). It started the so-called genomic revolution and the field of Personalized Medicine (PM), described as tailoring prevention, diagnostic methods and therapies based on patient’s genetic and lifestyle characteristics (3–5). Shifting away from the traditional “one size fits all” approach, PM offers more precise and effective treatments and better outcomes, leading to advantages for healthcare professionals and systems in terms of quality of care and sustainability (6, 7). The European Union (EU) issued several policies to foster the adoption of PM, such as digital innovation and interoperability, patients’/citizens’ engagement and healthcare sustainability (8, 9). Starting in 2015 with the definition of PM, the EU recognized the potential of PM and placed it at the forefront of its research agenda, supporting projects spanning the entire value chain of PM and promoting international collaborations through the International Consortium of Personalized Medicine (ICPerMed) (8, 9).

In the global networks, explored by specific Coordination and Support Actions funded by the EU Commission, the EU objectives in PM align with China (10), where the adoption and promotion of PM followed similar steps to the European scenario.

The Chinese attention to PM is testified by its inclusion in the 13th 5-Years Plan (2016–2020) and the following 14th 5-Years Plan (2021–2024), as well as in the Healthy China 2030 initiative. The country is committed to addressing the burden of non-communicable diseases and healthcare inequalities through substantial investments in multisector collaboration and innovation, particularly in cancer, promoting health education, raising awareness, improving early diagnosis, and enhancing treatment effectiveness. In addition, these plans address disparities in economic development and healthcare access across different regions of China, providing financial support, encouraging research and development, and creating a favorable regulatory framework (11, 12). Considering these similarities, the ICPerMed launched in 2020 the “Integrating China in the International Consortium for Personalized Medicine” (IC2PerMed) project, ultimately aiming at encouraging collaboration and integration in the network (8). Laying its methodology foundation in the “ICPerMed Vision for 2030” (13), the IC2PerMed project supports research, funding, and implementation of innovative PM approaches, with the overarching goal of establishing a preferential exchange platform between the EU and China (14). This comprehensive roadmap for PM implementation across both regions outlines action priorities to facilitate the exchange of best practices and address potential barriers, to deepen and promote alignment for the creation of a common ground for European and Chinese collaborations on PM.

## Materials and methods

**FIGURE 1:**
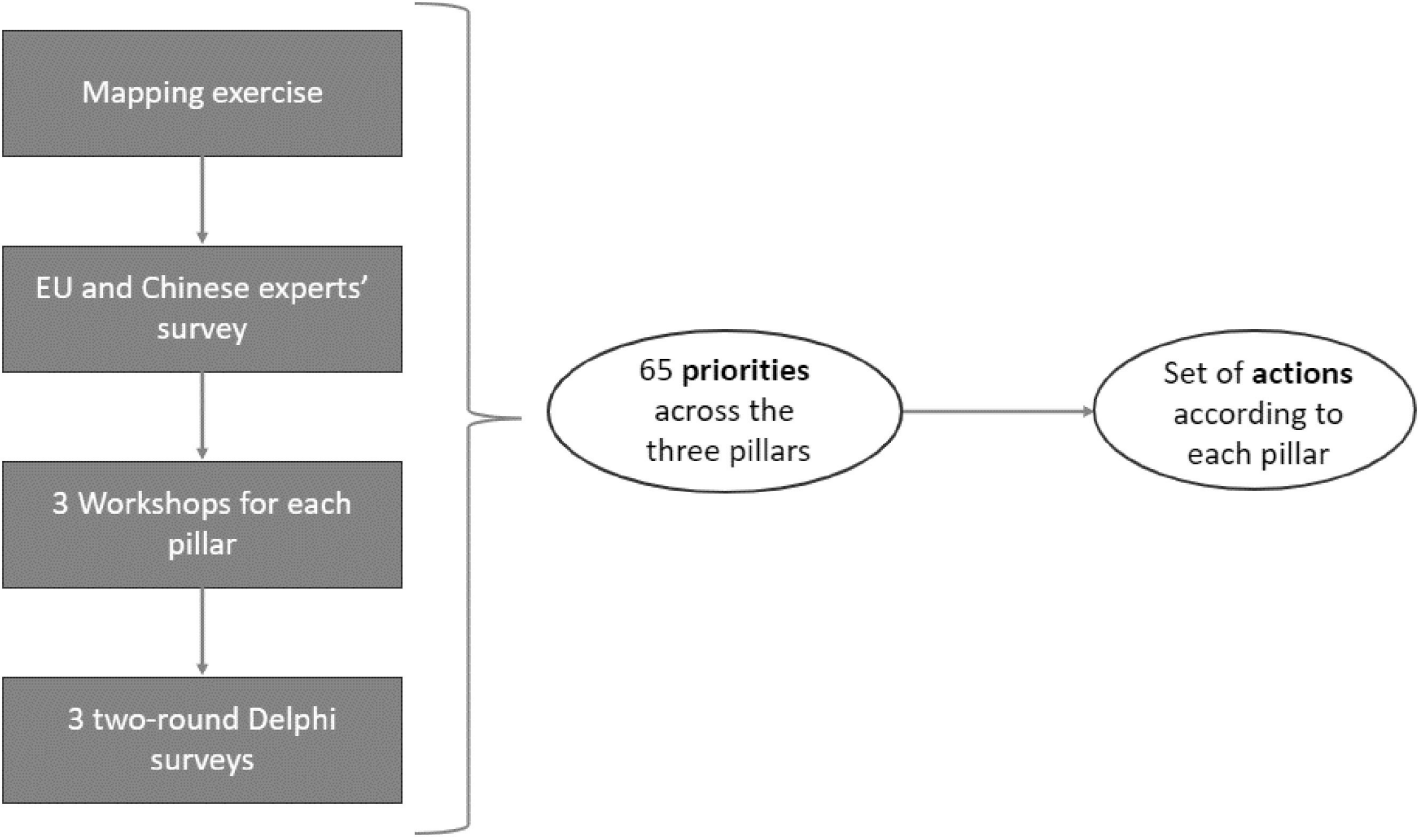
Methodological synthesis of the IC2PerMed Roadmap development process, This diagram outlines the multi-step methodology through which the IC2PerMed Roadmap’s set of actions was derived. The process began with a mapping exercise, followed by a survey involving EU and Chinese experts. Insights from these initial phases informed a series of three dedicated workshops–one for each pillar of the roadmap. Subsequently, three two-round Delphi surveys were conducted to consolidate expert consensus. This iterative and collaborative process led to the identification of 65 priorities distributed across the three pillars. These priorities then informed the formulation of a targeted set of actions for each pillar.

The ICPerMed approach has been used as a guiding framework during the whole conduction of the project. The activities were organized in four phases (Figure 1):

1. Mapping: identifying Chinese and EU PM-relevant policies, programs, stakeholders, actors and standards, to consider and involve in developments, envisioning benefits for healthcare ecosystems and for populations;
2. Expertizing: building upon exchange between experts’ domains for fostering actionable approaches;
3. Exemplifying: setting concrete practices of collaboration over a PM core theme (biobanks) for illustrating and inspiring research collaborations;
4. Engaging: creating solid bridges with European, Chinese and global key stakeholders, integrating Chinese stakeholders in ICPerMed and liaising with international peers.

The IC2PerMed Roadmap is the result of a three-step methodology:

- Mapping and experts’ survey
- Workshops and Delphi survey of experts
- Theme analyses and results synthesis into actions

### Mapping and survey of experts

A literature mapping explored the current landscape of PM implementation, relevant policies, projects, initiatives, standards and regulations in the EU and China. Findings were revised and expanded through a survey of 47 experts, from EU and China, exploring the main areas of interest and the international collaborations in place (10). The results were disseminated in scientific papers and publicly available deliverables (9, 14–17).

### Workshops and Delphi survey of experts

As reported in the introduction section, the project’s structure followed the classification laid out in the ICPerMed Vision for 2030 (12). The subsequent work was articulated in three pillars:

- Shaping sustainable healthcare
- Innovation and market
- Research and clinical studies in PM.

Three working groups (WGs) were established, each addressing the challenges of one of the three pillars, respectively:

- *Shaping sustainable healthcare*, focusing on developing awareness and empowerment of patients and citizens, education and training of healthcare professionals and healthcare sustainability;
- *Innovation and market*, focusing on Big Data and Information Communications and Technology (ICT) solutions and Bringing innovation to market;
- *Research and clinical studies in PM*, focusing on translating basic to clinical research and beyond and research funding.

Each WG organized three rounds of workshops to discuss preliminary priorities, identify potential gaps in literature results and up-to-date evidence on laws and guidelines, and bring important topics left out from the mapping into the discussion. Finally, nine workshops took place (18).

The preliminary results obtained in each of the three WGs were further evaluated in three Delphi surveys, one for each main pillar of discussion. The consensus was reached after two rounds for each Delphi survey, and some priorities were identified. The concepts of citizen empowerment and engagement are grounded in the International Association for Public Participation (IAP2) model of public participation (19).

### Delphi survey structure and consensus measurement

The Delphi method employed in the IC2PerMed project consisted of three separate two-round surveys, one for each of the three main pillars. In each round, experts were asked to rate the relevance of proposed priorities using a Likert scale from 1 (not relevant) to 5 (extremely relevant). Consensus was defined as ≥ 70% agreement among respondents for ratings of 4 or 5. The results of the first round informed the refinement and reduction of priorities in the second round. Outcomes were then synthesized to define a set of final priorities per pillar, which formed the basis for the roadmap actions.

The detailed methodology and the resulting list of 65 priorities were reported in four position papers (20–23).

### Theme analyses and results synthesis into IC2PerMed actions

For each pillar, a theme analysis on the identified priorities was performed, using the NVivo software (version 12). The final 65 priorities, derived from the position papers, were consequently merged into IC2PerMed actions that fed our roadmap, coding them according to the sub-topic they dealt with.

The results are presented qualitatively, reporting the coded sub-topics as bullet points.

The study protocol was approved by the Università Cattolica del Sacro Cuore Ethical Review Board (ID5249). The project structure, all derived outputs, and a graphical roadmap are available at www.icpermed.eu.

## Results

The actions of the IC2PerMed Roadmap are reported according to the three main pillars in the Table 1. For each pillar, the actions were further grouped according to the main priorities in each WG (Table 1). The detailed description of each action is reported below, for each of the three pillars.

**TABLE 1:** Overview of the IC2PerMed roadmap’s strategic actions across three pillars of personalized medicine (PM).

Pillar I: shaping sustainable healthcare			
*Empowering responsible citizens*	*Promoting a trained and up-to-date healthcare workforce*		*Fostering healthcare systems’ sustainability*
a. Health Literacy b. Research c. Public Trust d. ELSI	a. Education and ethics b. Collaborations c. Policies		a. Resources b. ELSI c. Evaluation d. Networks
**Pillar II: innovation and market**			
***Bringinginnovationtomarket***		***Adopting Big Data and ICT solutions***	
a. Cost-effectiveness b. Needs assessment c. Principles and guidelines d. Perspectives		a. Data exchange b. Privacy, security, and trust c. Standards	
**Pillar III: research and clinical studies in PM**			
***Fosteringresearchfunding***		***Translating basic clinical research and beyond***	
a. Patient needs b. Value chain c. Synergies		a. Omics sciences b. Data and standards c. Collaborations	

### Pillar 1: shaping sustainable healthcare

*I-Empowering responsible citizens*

a. Health literacy

Promoting health literacy is a prerequisite for better citizens’ and patients’ engagement and empowerment. The growing significance of digital technologies and the pivotal role they play in facilitating the engagement process underscore the necessity to enhance digital literacy. Given the advancement of genomics and the widespread use of predictive genetic/genomic testing, informing citizens and patients could give them greater awareness about their health trajectories. The impact of healthcare professionals’ literacy should be considered, as they are a proxy for public engagement in the self-management of health and disease.

b. Research

Fostering needs-assessment research and communication activities in the field of citizens’ and patients’ education related to PM could lead to more effective empowerment of citizens and patients alike.

c. Public trust

Scientific research, public organizations and private institutions are key innovation actors in PM. Sustaining public trust and collaborations between different institutions nationally and internationally is the drive for healthcare transformation and public health promotion. In addition, public trust should be fostered and strengthened to protect patients’ rights through clear data governance in accordance with the Helsinki Declaration and the General Data Protection Regulation (GDPR), implementing technical solutions to safeguard cyber security, citizens and health practitioner engagement, and developing comprehensive consent procedures where needed.

d. Ethical challenges

A valid set of values and ethical principles should focus on the economic challenges and the inequality burden.

*II-Promoting a trained and up-to-date healthcare workforce*

a. Education and ethics

Improving healthcare professionals’ literacy and expertise, valuing integrity and ethics, could help foster PM. Research aimed at identifying effective methods should be promoted.

b. Collaborations

The future of healthcare professionals’ training relies on multidisciplinary collaborations. Fostering collaborations between professionals from different specialties and between professionals and stakeholders while establishing more partnerships among countries to facilitate sharing of best practices.

c. Policies

Literacy in PM among healthcare professionals is an emerging focal point in national governmental strategies, policies, and agendas.

*III-Fostering healthcare systems’ sustainability*

a. Resources

A better allocation of resources on PM can foster the sustainability of health systems. In particular, the identification of a large investment stream for long-term data storage is a fundamental prerequisite for implementing PM strategies. Investment priorities for product and process innovation should be defined, considering the relationship between results and costs by identifying new payment models for public reimbursement.

b. ELSI & Costs

Ethical, Legal, and Social Implications (ELSI) and associated expenses must consistently factor into policy formation, assessment, and the governance of technological advancements in PM.

c. Evaluation

Health technologies are evolving rapidly and the translation of new discoveries underpins innovation and quality of care. Therefore, a system of continuous assessment of technologies and processes already in use and a change of perspective in Health Technology Assessment (HTA) is needed to integrate end-user perceptions into the innovation process. This would ensure greater effectiveness and usability.

d. Networks

Multidisciplinary and cross-sectoral collaborations for PM can promote the sustainability of health systems. Public-private partnerships and international networks should be valued for sharing experiences and promoting and evaluating best practices and progress in PM.

### Pillar 2: innovation and market

*I- Bringing innovation to market*

a. Cost-effectiveness

The application of personalized diagnostics and therapeutics should be geared toward lowering economic costs and barriers to market uptake. Regarding diagnostics, promoting research in PM aimed at more appropriate use of diagnostic tools (avoiding overuse, overdiagnosis and overtreatment) could lead to optimal use of resources in the field of prevention and, consequently, an increase in the value of healthcare. Health insurance providers should extend their coverage to innovative and high-value PM solutions, and reimbursement of services should be promoted or attempts should be made to reduce barriers to reimbursement. In implementation processes, economic, cost-effectiveness, and relative value analyses should consider both social and health budgets and non-optimal resource use in the system.

b. Needs assessment

Considering the epidemiological scenario, new solutions on the market must emphasize maximizing health outcomes for patients. Early, intensive, coordinated and continuous dialogue among all PM stakeholders is needed to foster process optimization and accelerate the acquisition of new technologies and tools.

c. Principles and guidelines

The PM actors should follow shared principles and universal data sharing and exchange guidelines. Innovations that aim for higher therapeutic value should be rewarded. Social value assessment should be systematically applied.

d. Perspectives

Stakeholders stimulating innovation should take a holistic and long-term perspective on the balance sheet. The interconnection and mutual dependence between diagnostic and therapeutic innovations and the potential for inappropriate use/overuse must be taken into account.

*II-Adopting Big Data and ICT solutions*

a. Data exchange

To promote PM, Big Data must be analyzable, comparable and interoperable across borders. The need emerges to carefully identify the type of information to be retained, increasingly favoring those related to health outcomes rather than information with no proven clinical or management value. To facilitate data exchange procedures, greater cooperation between academia, healthcare systems (including providers and payers), and industry would be advisable.

b. Privacy, security, and trust

Data security measures are a priority in developing new ICT solutions, which are crucial at the global level and not only focusing on high-income countries. Social and cultural differences between Europe and China should also be considered when it comes to public trust in government and state authorities, trying to reach a common understanding of shared challenges within PM. Involving the public can enhance awareness about data sharing benefits, its extended purposes, anonymization, privacy risks, security, private sector involvement, personal data protection, and foster frameworks aligned with societal agreement.

c. Standards

In the PM field, it is essential to study solutions aimed at effectively combining data from different sources (genetics, clinical data) and regions, focusing on their standardization for effective usage. Standards for data use should be adopted and implemented, also with a view to establishing common policies and global efforts for cross-border data sharing.

### Pillar 3: research and clinical studies in PM

*I-Fostering research funding*

a. Patient needs

Funding agencies should tailor investments to the needs of patients. There is a need to promote the voice of patients (and caregivers) at all stages of PM research, from co-designing research projects to advisory roles and enhancing educational initiatives to improve the scientific literacy of patients and researchers. Defining unmet needs and potential incremental innovation could help in laying the groundwork for new international collaborations.

b. Value chain

Investments, playing an important role in the entire value chain, are needed from basic science to implementing PM in healthcare. Funders, both public and private, act as the first filter on the prioritization of resource allocation which should be done responsibly. Furthermore, adequate investments are crucial in the research translation system.

c. Synergies

Establishing synergies between funders and the research community is the first step in implementing PM as a community. Implementing the exchange of researchers through mobility funding programs could promote collaboration and knowledge sharing between different countries and foster data sharing. Collaborations between funders should be established to align on research themes and fund larger, bold, cutting-edge projects that enable risk sharing.

*II-Translating basic clinical research and beyond*

a. Omics sciences

Omics sciences are fundamental to the development of PM. Phenotyping patients, following defined standards, could identify similar patients. Besides genomics, applications of different omics sciences and technologies should be promoted and used to identify biomarkers suitable for PM. Innovative methods that have shown great promise in the PM field, including induced pluripotent stem cell and organ-on-chips models, should be evaluated and adopted, valuing international partnerships.

b. Data and standards

Standardizing approaches, including controlled access models for data sharing and clinical trials, may facilitate their implementation and help in patient stratification. Patient stratification in non-genetic/complex diseases would benefit from research programs on machine learning algorithms. Furthermore, using specific use cases could help in the development of common international standards and tools for research. Exchanges and dialogue between regulatory agencies should be promoted to overcome regulatory problems in PM, particularly on benefit-risk relationships.

c. Collaborations

It is important to support non-profit foundations and funding agencies to promote international collaborations, especially in oncological care and rare diseases. Establishing specific funding programmes and operational frameworks for public-private collaboration can facilitate academic and industrial access to biological samples and data for research purposes. It is necessary to facilitate and strengthen the dialogue with regulatory and HTA agencies, companies, and academic entities to gain a clear vision regarding outcomes researched and identify the most appropriate research methods to investigate PM, ensuring patient safety and adapting to the characteristics of study populations.

## Discussion

The IC2PerMed roadmap illustrates strategic actions aimed at fostering international collaboration between China and the EU in the PM realm. These actions are intended to facilitate collaborative initiatives and enhance the optimization of research and developments, using the three pillars feeding the working groups’ activities as cornerstones.

Closer cooperation between the two sides can lead to synergies and gains for both. The alignment of research efforts and promotion of common research initiatives in science and technology could lead to reduction of redundancies and improved use of available monetary, technological and human resources. Generating substantial evidence can often be challenging due to limited data availability. This is particularly frequent for patients affected by some conditions, or undergoing personalized interventions, that might not generate enough data. Partnerships between institutions working in the same field can facilitate the collation of available evidence by pooling available data. On the other hand, collaborations between diverse entities can lead to reciprocal benefits, e.g., feedback on data reproducibility to basic research institutions while enabling translational institutions to gain insights and preliminary findings from closer collaborations. In this context, clusters that bring together different entities and foster cross-field cooperation play a crucial role in facilitating such joint efforts. The current high fragmentation of the sector calls for concerted efforts toward integration under common guidelines.

Engaging with all stakeholders is essential to incorporate diverse perspectives and ensure effective policy development (17). The IC2PerMed roadmap could orientate the efforts of policymakers, industry, healthcare professionals and citizens.

Policymakers often struggle to keep up with innovation, especially in high-transformative fields like PM. Policymakers play a central role as the primary recipients of countries’ assistance and research priorities, wielding significant influence in the formulation and implementation of healthcare policies. The regulatory landscape is heterogeneous and fragmented in the EU and China, often relying on *ad hoc* initiatives and projects (9). The speed at which innovation proceeds is often incompatible with the times of policymaking, leaving much room for the introduction of unregulated technologies into practice, sometimes with negative effects (9). In healthcare, normative gaps can expose people’s health and well-being to unanticipated risks. Therefore, emerging technologies and processes should be carefully evaluated before they are adopted (24). Significant issues can arise, possibly related to the unintended effects of adopting some technology or concerning the data generated by it. If not stored safely and anonymously, data leakages or hacker attacks can lead to data theft, with several unwanted consequences that are particularly negative in the case of genomic data (25).

Facilitating the exchange of health data among different institutions, even from different countries, is a priority in the EU, that is working for the creation of the European Health Data Space to address health-specific challenges to electronic health data access and sharing, focusing on interoperability and cross-border sharing. More federated data infrastructures (as fostered by the 1 + Million Genomes Initiative, which has led to the Beyond 1 + Million Genomes and the Genomic Data Infrastructure projects) will also contribute to this goal (26–30).

Big Data raises several issues, including personal data ownership and protection, skill gaps in labor markets and an emerging new digital divide (31). Hence, policies in this field are fundamental for regulating these aspects (31). Regarding data protection, notable distinctions exist between Europe and China.

Europe’s regulation on data, the General Data Protection Regulation (GDPR), places significant emphasis on obtaining explicit consent or relying on legal exemptions for utilizing health data. In cross-border collaborations with third countries, such as China, data transfers necessitate adherence to the same level of data protection as enforced in the EU (32). Conversely, China has implemented a comprehensive data protection framework, encompassing laws such as the Data Security Law, Cybersecurity Law, and Personal Information Protection Law (33). These regulations are primarily oriented toward national security, public interest, and safeguarding fundamental data. Moreover, China has specific regulations, such as the Measures for Managing Scientific Data, governing the transfer of scientific data to designated data centers. These measures ensure the confidentiality and security of data, with a particular focus on prohibiting the disclosure of sensitive information (34–36).

Along regulations like GDPR and the Helsinki Declaration, a key opportunity lies in enhancing cross-border regulatory interoperability. Mutual recognition of ethical standards–such as data consent procedures, ethical review protocols, and data transfer regulations–could reduce friction and foster trust. Establishing joint ethical working groups or harmonization task forces may provide the foundation for a common ethical framework in international PM research.

The contrasting approaches between the two parts in data protection stem from their respective regulatory frameworks, varying consent requirements, and targeted legislation addressing different aspects of data protection and governance and promoting partnerships to facilitate data sharing and the creation of collaborative datasets (28, 37).

However, new solutions and advanced technologies, even if promising better outcomes for patients, can come at a high cost, hindering the implementation process and healthcare systems’ sustainability (38, 39). Some solutions have been proposed, from checklists and instruments to measure PM solutions’ utility to performing economic evaluations that would ensure an intervention’s cost-effectiveness, like HTA and Health Impact Assessment (HIA) (40, 41). Nonetheless, need-assessment and integration of end-users’ perspectives seem to be neglected.

For PM to reach its impact on patients’ health and well-being, translation of discoveries and communication across the continuum of research is required (42). The development of preclinical and clinical model of disease benefits from the integration of ‘-omics’ data, useful to define molecular profiles. (43). In order to standardize the process, identify biomarkers and correctly collect data, international consensus should be reached (43). Developing new clinical trial designs including these innovative instruments and approaches contribute to the outcome research and the evaluation of interventions (43). Cross-sectional research and collaboration should be supported by suitable funding mechanisms, valuing the existing instruments or promoting the establishment of new ones (44). As per the mapping of major funding agencies and stakeholders in Europe and China in IC2PerMed’s deliverable D1.2, great similarities can be found in the structure of funding mechanisms and areas of interest (45).

Concerning industry and universities, which are the main actors in PM research and innovation in the EU and China (17), the results refer to the importance of investments of translating research into the market and partnerships between industrial partners and academia. Collaborations hold large economic potential, where global leaders can extend their reach and value chains to new markets. Both parties should pursue partnerships, collaborations and workshops to communicate and interact. Nonetheless, bridging the translational gap is crucial, and ensuring that universities and industries communicate will help address issues such as the skills mismatch (46, 47).

Implementation of the roadmap should be accompanied by a robust monitoring and evaluation (M&E) strategy. This may include periodic stakeholder consultations, scorecards to track action uptake, and impact assessments. Evaluation should not only measure outputs (e.g., number of actions implemented), but also outcomes (e.g., increased access to PM services, improved diagnostic accuracy, or cost-effectiveness gains).

To support roadmap uptake, the alignment with existing policy instruments in both the EU and China is critical. The roadmap’s strategic actions can serve as a blueprint for integrating PM into national healthcare agendas. To track progress, the policymakers could take into consideration the adoption of indicators, such as, for example: number of national programs adopting specific roadmap actions; integration of PM in clinical guidelines; regulatory frameworks updated to include PM-specific provisions; patient participation metrics in PM trials. These indicators could be monitored jointly through EU–China bilateral review mechanisms.

As a more concrete example, the implementation of pharmacogenomic testing for dihydropyrimidine dehydrogenase (DPYD) variants prior to fluoropyrimidine treatment (e.g., 5-fluorouracil) could serve as a measurable goal of PM uptake. These variants are associated with severe toxicity and early death in patients undergoing chemotherapy. Despite established clinical guidelines, uptake remains limited. Through targeted awareness campaigns, infrastructure investment, and regulatory incentives, a realistic target could be to increase DPYD testing implementation rates from current baseline levels (approx. 20%–30%) to at least 70% by 2030 across EU and Chinese oncology centers. This example illustrates how the roadmap’s strategic actions can be translated into tangible, life-saving outcomes for patients (48).

To implement PM in clinical and public health practice, both patients and health professionals should be aware of the possibilities offered and how to make the most of them (6, 20). Given the need for informed, empowered, engaged and responsible citizens, there is a need to deepen digital literacy, knowledge of health data, public trust in institutions and easily accessible, reliable and understandable sources of medical information. Informed, accountable and empowered health service providers are also essential. Establish a normative framework for the exploitation of health information and research results in PM should be implemented for clinical practices (49). Multidisciplinary collaborations improve the involvement of citizens in decision-making, promoting the creation of advocacy groups to represent the needs and perspectives of citizens and patients with policymakers (49). Patients/citizens advocacy groups should collaborate with healthcare professionals and policymakers to improve health literacy and involvement in research, as well as to identify citizens’ needs and sources of lack of trust (50). It should be noted that the bilateral efforts on standardization in PM will benefit the whole field and allow health challenges to be tackled globally in a concerted manner.

Data scientists emerge as core members of multidisciplinary tumor boards, especially in low-resource settings. Their role in bridging omics data, clinical interpretation, and regulatory constraints is increasingly seen as essential to sustainable PM implementation (51).

PM should consider both social and health budgets and non-optimal resource use in the system. This is consistent with calls from global experts to ensure that precision medicine is not only scientifically robust but also economically valuable. Realizing PM’s clinical potential requires alignment of reimbursement models, clinical utility standards, and regulatory frameworks (52).

Looking ahead, the roadmap outlined in this work could be further enriched by considering the emerging technological paradigms that are shaping the future of hyper-personalized medicine. In this regard, the vision proposed by Tan et al. (53) offers a compelling complement to the policy and collaboration framework we present (53). Their work highlights how advanced technologies–such as Internet of Things (IoT) devices for real-time lifestyle and environmental monitoring, 6G connectivity for instantaneous data transmission, and the potential role of Artificial General Intelligence and Quantum Computing in processing complex health datasets–can transform the implementation of Personalized Medicine (PM) into a continuous, adaptive process. These tools could serve as enablers for many of the roadmap’s proposed actions. For example, the use of health dashboards fed by personal IoT devices, as discussed by Tan et al., represents a tangible mechanism for enhancing digital health literacy and empowering citizens to actively manage their health (Pillar 1). Similarly, the emphasis on data interoperability and standards in our roadmap (Pillar 2) resonates with Tan et al.’s call for robust technological infrastructures that can support real-time, multidimensional data integration. Finally, the concept that health is “beyond genetics” supports the expansion of PM research beyond omics into integrated models that capture the interplay between genetic, lifestyle, and environmental determinants of health (Pillar 3). Embedding these forward-looking perspectives into the roadmap helps frame a dynamic path from present policy and collaboration strategies to future-ready, technology-enabled PM ecosystems.

The results of this work should be considered in light of some strengths and limitations. To the authors’ knowledge, this is the first attempt to present a comprehensive roadmap addressing multiple issues in the field of PM, with a specific focus on the collaboration with China. This document offers a tool for policymakers, highlighting emerging issues and experts’ considerations in the field. In addition, this roadmap includes other relevant stakeholders in the field of PM, adding value to its content.

The methodology used, particularly careful to intertwine the scientific literature, institutional sources and the opinion of experts, led to building a roadmap where each step was carefully evaluated and validated. This helped guarantee the quality and reliability of the results. In addition, the roadmap provides indications on aspects of PM that want to be as comprehensive as possible of the current scenario and challenges. However, arising from the international comparison of two realities, the European and the Chinese, which do not completely overlap in culture, political strategies, health systems, and technological advancement, it is possible that some aspects sound less specific compared to others. Furthermore, it is possible that some aspects relevant to PM have been neglected because of the adoption of as “perspectives” identified in the ICPerMed Vision for 2030. However, some additional limitations should be acknowledged. First, the absence of a structured evaluation phase represents a significant constraint, as it hinders the systematic assessment of the actual implementation and impact of the proposed actions. While the roadmap suggests key performance indicators and outlines a potential monitoring approach, a concrete and operational evaluation framework is still lacking. Furthermore, the division of content into distinct position papers, although useful for clarity and focus, may give rise to a perceived fragmentation, potentially obscuring the overarching coherence of the roadmap and its strategic vision. Moreover, the roadmap aims to identify common ground and shared priorities between the EU and China, rather than providing an exhaustive comparative analysis of each country’s specific strengths and limitations. This choice reflects the methodological decision not to cluster Delphi responses by the country of origin of the participating experts. While this approach supported a higher response rate and enhanced engagement with the project, it also ensured the anonymity of the data collected. As a result, it was not possible to explore country-specific divergences or tailor recommendations accordingly, which may have limited the granularity of the analysis.

## Conclusion

PM has the potential to disrupt the medical field bringing major improvements for the benefit of public health. In doing so, PM affects citizens, patients, their families, and communities, at all levels of the entire healthcare system.

The development of PM requires concise action across universities, industrial partners, and national governments and urges for synchronous development on a global scale. To do so, the alignment of European and Chinese efforts, finding common ground across cultural, social, and language barriers can enhance public health efforts in applying PM strategies internationally.

Although different countries may have different perspectives because the specific national agenda may differ, PM acts as a collector of converging interests by acknowledging the best care for citizens and patients at its center. The IC2PerMed roadmap represents an instrument for PM implementation, tackling the modern challenges of medicine and healthcare pragmatically in a list of doable actions. Implementing PM in a transversal way and welcoming new discoveries while considering sustainability holds the potential to have a revolutionary impact on healthcare, but this will require a concerted effort.

## Data Availability

The original contributions presented in the study are included in the article/supplementary material, further inquiries can be directed to the corresponding author.
